# Defining rules for cancer cell proliferation in TRAIL stimulation

**DOI:** 10.1038/s41540-019-0084-5

**Published:** 2019-02-15

**Authors:** William Deveaux, Kentaro Hayashi, Kumar Selvarajoo

**Affiliations:** 1 École nationale d’ingénieurs de Brest, Brest, France; 2 Institute for Advanced Biosciences, Keio University, Tsuruoka, Japan; 3 Biotransformation Innovation Platform (BioTrans), Agency for Science, Technology & Research (A*STAR), Biopolis, Singapore

## Abstract

Owing to their self-organizing evolutionary plasticity, cancers remain evasive to modern treatment strategies. Previously, for sensitizing tumor necrosis factor-related apoptosis-inducing ligand (TRAIL)-resistant human fibrosarcoma (HT1080), we developed and validated a dynamic computational model that showed the inhibition of protein kinase (PK)C, using bisindolylmaleimide (BIS) I, enhances apoptosis with 95% cell death. Although promising, the long-term effect of remaining ~ 5% cells is a mystery. Will they remain unchanged or are they able to proliferate? To address this question, here we adopted a discrete spatiotemporal cellular automata model utilizing simple rules modified from the famous “Conway’s game of life”. Based on three experimental initializations: cell numbers obtained from untreated (high), treatment with TRAIL only (moderate), and treatment with TRAIL and BIS I (low), the simulations show cell proliferation in time and space. Notably, when all cells are fixed in their initial space, the proliferation is rapid for high and moderate cell numbers, however, slow and steady for low number of cells. However, when mesenchymal-like random movement was introduced, the proliferation becomes significant even for low cell numbers. Experimental verification showed high proportion of mesenchymal cells in TRAIL and BIS I treatment compared with untreated or TRAIL only treatment. In agreement with the model with cell movement, we observed rapid proliferation of the remnant cells in TRAIL and BIS I treatment over time. Hence, our work highlights the importance of mesenchymal-like cellular movement for cancer proliferation. Nevertheless, re-treatment of TRAIL and BIS I on proliferating cancers is still largely effective.

## Author summary

Cancers are evasive to even the most modern state-of-the-art treatment strategies. Although numerous studies, on distinct drug treatments, have shown large portions reaching cell death, the long-term effect of the remaining resistant cells is a mystery. Will they remain unchanged or are they able to proliferate? To address this question, an international team from Japan, France, and Singapore, led by Kumar Selvarajoo, has developed a spatiotemporal cellular automata model that tracks cancer proliferation in time and space, for cancers untreated and treated with potential drug targets (TRAIL and TRAIL plus BIS). Their model, based on setting rules for cancer proliferation, predicted that mesenchymal-like cellular movement, rather than actual cancer numbers or size, is key for cancer proliferation. The results were subsequently validated experimentally on human fibrosarcoma.

## Introduction

Cancer cells are highly heterogeneous, not only in genetic variability between individual cells, but also in their morphology, intracellular constituents, and molecular expression dynamics.^1^ Recent works have shown that cancers can evolve non-genetically and are able to make the epithelial-mesenchymal transition (EMT), providing with high motility to form metastasis of surrounding and other far-from-connected tissues.^2,3^ It is, therefore, conceivable why most, if not all, invasive and non-invasive treatment strategies, based on the predominant “average cell” (all cells being equal) approach, to tackle and control the complexity of cancer succumb to cell proliferations. To understand the complexities of dynamic cancer response, and to regulate them successfully, experimental approaches alone are insufficient. Numerous mathematical and computational models have been developed to interpret and predict the dynamics of cancer cell survival/proliferation and to identify targets for enhancing apoptosis.^4,5^ Lavrik^6^ has edited an excellent book that provides a succinct review on the numerous statistical, Boolean and kinetic models developed to understand cancer cell apoptosis.

Tumor necrosis factor-related apoptosis-inducing ligand (TRAIL), a proinflammatory cytokine produced by our immune system, has shown promising success in controlling cancer threat, owing to its specific ability to induce apoptosis in cancers while having nominal effect on normal cells.^7,8^ Nevertheless, several malignant cancer types remain non-sensitive to TRAIL. A notable example of TRAIL-resistant cancer is HT1080, where on average, only 40% of cells respond to treatment.^9,10^ In a previous work, we developed an ordinary differential equation-based kinetic model to track the cell survival and apoptosis signaling, through MAP kinases/NF-κB and caspase -8/-3 dynamics, respectively, in TRAIL-stimulated HT1080.^10^ To sensitize HT1080 to TRAIL treatment, we performed several in silico intracellular target suppression, and evaluated the overall cell survival ratios. The model indicated protein kinase (PK)C inhibition, together with TRAIL, is the best treatment strategy that could induce 95% cell death. To confirm this result, we subsequently performed experiments using the PKC inhibitor, bisindolylmaleimide (BIS) I in HT1080 and another TRAIL-resistant cell line (human adenocarcinoma HT29) and showed over 95% cell death in both cell lines.^11^ Despite the use of the “average cell” modeling approach, the simulations accurately predicted the experimental outcome. Although the finding holds promise for cancer treatment, the long-term fate of the remaining (~ 5%) HT1080 remains unknown and may be difficult to predict using popular current modeling approaches including our previous models.^12,13^ Will they be quiescent, or are they able to self-organize and proliferate? Hence, despite hugely challenging, we require alternative approaches that could integrate cell signaling outcomes with macroscopic cancer evolution considering cell-to-cell contact.

The investigation of dynamic complexity, or self-organization in biology, requires “integrated” knowledge gained from diverse disciplines. There have been numerous computational efforts to understand self-organization, where a large proportion utilizing continuous differential equation approaches.^14,15^ These approaches require deep understanding on the underlying mechanisms, and the appropriate parameter values for successful modeling. Here, we needed a simpler method as most signaling, transcriptomics or evolutionary details of cancer cell proliferation are unknown.

Cellular automata (CA) is a discrete computational methodology that utilizes user defined simple rules to predict the behavior of an automaton or cell in time, space, and state.^16^ The rules adopted can be based on physical laws or simple imagination, and can be tailored to match experimental reality. Owing to the iterative process of trying different rules for the convergence of the intended outcome, one could be able to identify a set of rules that best represent the underlying mechanism. The most popular CA is Conway’s game of life.^17^ By using self-defined simple rules, Conway produced diverse and complex self-organizing patterns. Subsequently, other works have demonstrated its attractiveness in various disciplines, including biology.^18,19^

Here, we adopted the CA approach to predict the outcome of HT1080 under three experimental conditions; (i) untreated, (ii) treatment with TRAIL only, and (ii) treatment with TRAIL and BIS I. As details on cancer cell proliferation and self-organizing response after any drug treatment is scanty, we wanted to investigate whether (i) simple rules, such as CA rules, can be used to track cancer cell proliferation and, (ii) models developed using such rules can be experimentally tested.

## Results

### Spatiotemporal CA model (model A)

We developed a CA model (Methods), initially using rules and related parameters from Conway’s game of life:

i. Any cell with less than two live neighbors dies, caused by under-population.
ii. Any cell with two or three live neighbors lives on to the next generation.
iii. Any cell with more than three neighbors dies, caused by overcrowding.
iv. Any dead/empty cell with three live neighbors becomes live cell as by reproduction (division).

Although the rules, owing to its abstractness or oversimplification, may not be sufficient to model cancer self-organization successfully, nevertheless, they can generate complex self-organizing spatiotemporal patterns that have been explored in numerous scientific fields.^20^ We simulated to see how the automata will evolve in a simulated dish for three experimental conditions: (i) untreated HT1080 (WT), (ii) HT1080 treated with TRAIL (TRAIL), and (iii) HT1080 treated with TRAIL and PKC inhibitor BIS I (TR + BIS) based on the actual cell numbers after initial treatment. These conditions were taken from our previous experimental work in a dish,^11^ where we estimated the actual cell numbers for each condition to initialize the CA model. WT had an average of 1 × 10^5^ cells, TRAIL had 6 × 10^4^ and TR + BIS had 5 × 10^3^ cells after 24 h treatment.

**Fig. 1:**
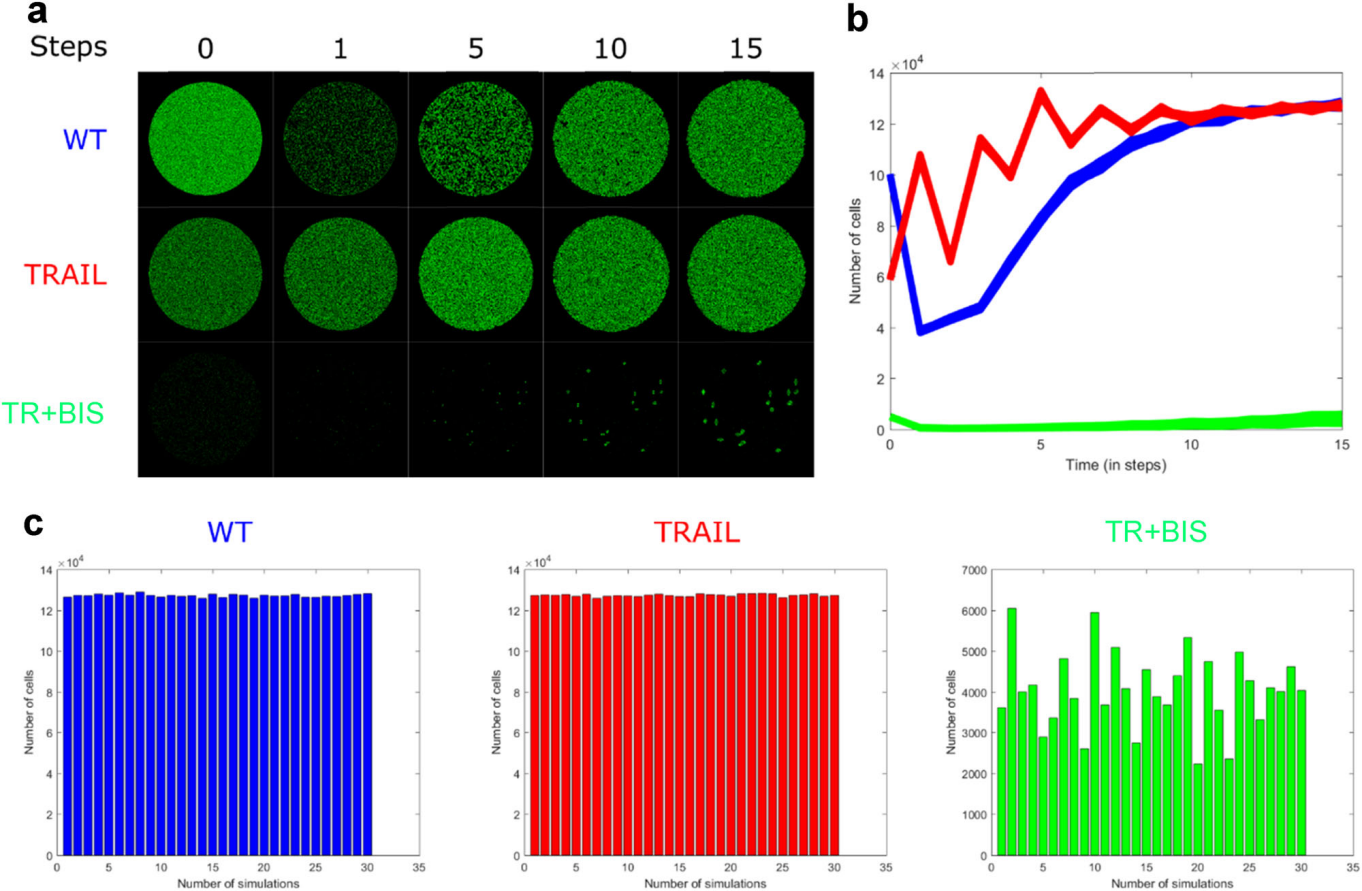
Simulations of spatiotemporal evolution of HT1080 cells using CA model A. **a** 2-D top view of the simulations for 15 time steps, **b** the total numbers of cells in time, **c** the number of survival cells at end time ( = 15) for 30 simulations. Untreated (WT), TRAIL stimulated (TRAIL), and TRAIL and BIS I treated (TR + BIS)

In other words, we run the CA model with Conway’s game of life in three initial conditions determined by the number of cancer cells in unstimulated (WT), after 24 h treatment using TRAIL alone (TRAIL), and TRAIL plus BIS I (TR + BIS). The model was simulated for 15 discrete arbitrary time steps and repeated 30 times to check whether the random orientation of cells for each run at initial time ( = 0) will have any significant variable outcomes (Fig. 1). For WT (high initial cell numbers), we observed the number of HT1080 cells decreased significantly for the first time-step, owing to overcrowding (Rule iii), but quickly recovered and increased rapidly with time (Fig. 1a, b, blue). For TRAIL (moderate initial cell numbers), the simulations, although fluctuating, shows a general increase with time and converged to similar cell numbers with WT by 15 time steps (Fig. 1a, b, red). Note that the variability between each of the 30 simulations were low for both WT and TRAIL (Fig. 1c, blue & red). Contrary, TR + BIS (low initial cell numbers) showed stable population of cells in time, despite relatively high variability between the simulations (Fig. 1a–c, green). Note that in some runs, the actual cell numbers are even decreased from time 0 (Fig. 1b, c, green). In other words, the CA Model simulations indicate that, if all the initial cell numbers are low (TR + BIS) and fixed in their location (immovable), the cell proliferation capacity will remain low. However, if the initial numbers are moderate (TRAIL) or high (WT), the cells will continue to proliferate over time. Thus, according to our simulations, TR + BIS may successfully control the progression of HT1080 in cell cultures over time.

### CA model with distinguishing E & M cells (model B)

It is now generally appreciated that EMT occurs during cancer cell progression and metastasis.^2,3^ The EMT is a central process of embryogenesis or normal cell development, in which epithelial (E) cells reach fibroblast-like mesenchymal (M) state. In brief, the fairly round shaped E cells are connected by cell–cell adhesion, facilitating cell layer formation and remain largely static.^21,22^ Unlike the E cells, the thin and long shaped M cells are not bounded to other cells and are free to move across a medium.^23^ It is also known that, under stressed conditions, E cells can transform into M cells, a process that is referred to as EMT.^24^

**Fig. 2:**
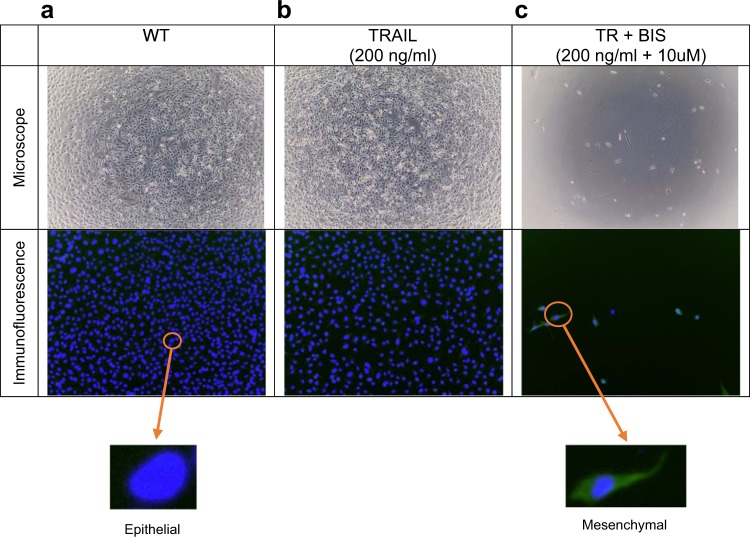
Microscope and immunofluorescence images of HT1080. Cells were imaged by microscope (top) and Cytell imaging system using dyed nucleus in blue (bottom). Based on the cell morphology and the intensity of green anti-Vimentin antibody fluorescence (Methods), cells can be classified as epithelial or mesenchymal (see insert). **a** WT, **b** TRAIL, and **c** TR + BIS

It is notable to mention here that HT1080 are fibrosarcoma cell line derived from a mesenchymal lineage, and hence, often considered not to exist in the epithelial stage. However, recent works have indicated the occurrence of mesenchymal-to epithelial transition in fibroblast cells.^25^ To investigate this, we performed experiments on HT1080 cells, in 48 plated wells (Methods), left untreated, stimulated with 200 ng/mL of TRAIL, and pre-treated with 10 μm of BIS I prior to TRAIL stimulation (200 ng/mL). Notably, the experiments reproduced previous observations of ~ 60% and 5% cancer cell survival (compared with unstimulated) after 24 h for the TRAIL and TR + BIS treatments, respectively (Fig. 2a–c, top panels).

When the morphology of the HT1080 cells was checked on the microscope, they mainly appeared to be made up of E cells for the unstimulated and TRAIL-stimulated condition, whereas TR + BIS treatment showed visible M cells (Fig. 2a–c, top). To confirm this observation, we used anti-Vimentin antibody with our HT1080 cell culture to track its fluorescence (Vimentin, an intermediate filament protein specifically expressed in M cells, is often used as a marker for mesenchymal cells^26–28^). We noticed that untreated HT1080 cell culture did not show any Vimentin fluorescence, indicating large majority of E cells (Fig. 2a, bottom). However, when we investigated the fluorescence for TRAIL, and TR + BIS-treated cells, we observed fluorescence for certain cells for the former and majority for the latter (Fig. 2b, c, bottom). Based on the fluorescence intensity, we estimated that the number of M cells in HT1080 was low for untreated (~ 2%) and TRAIL treated (~ 10%), but significantly higher for TR + BIS treated (~ 95%) (Fig. S1A). Thus, from these data, we gather that TR + BIS treated remnant cells have largely undergone EMT in a plate.

It is now conceivable that the rules for the automaton in our model should be able to reflect the generalized cancer cell proliferation, considering the EMT. Thus, rules (ii) and (iv) were modified and two additional rules (v and vi, see below) to distinguish E and M cells in the HT1080 population were included. The overall rules are as follows:

i. Any E cell with less than two live neighbors dies, caused by under-population.
ii. Any E cell with two or three live neighbors become M cells on to the next generation.
iii. Any E cell with more than 3 neighbors dies, caused by overcrowding.
iv. Any dead/empty cell with three live neighbors (E or M) becomes live cell (E or M) as by division.
v. Any M cell is able to move randomly to an empty cell on to the next generation.
vi. Any M cell that is unable to move becomes an E cell on to the next generation.

The rationale for choosing rule (v) is based on the fact that M cells are mobile,^21^ hence, they are allowed to move anywhere in the lattice randomly. For rule (vi), it is now known the reverse of EMT, the mesenchymal-to-epithelial or MET, is also a possibility when cells become crowded.^29^

**Fig. 3:**
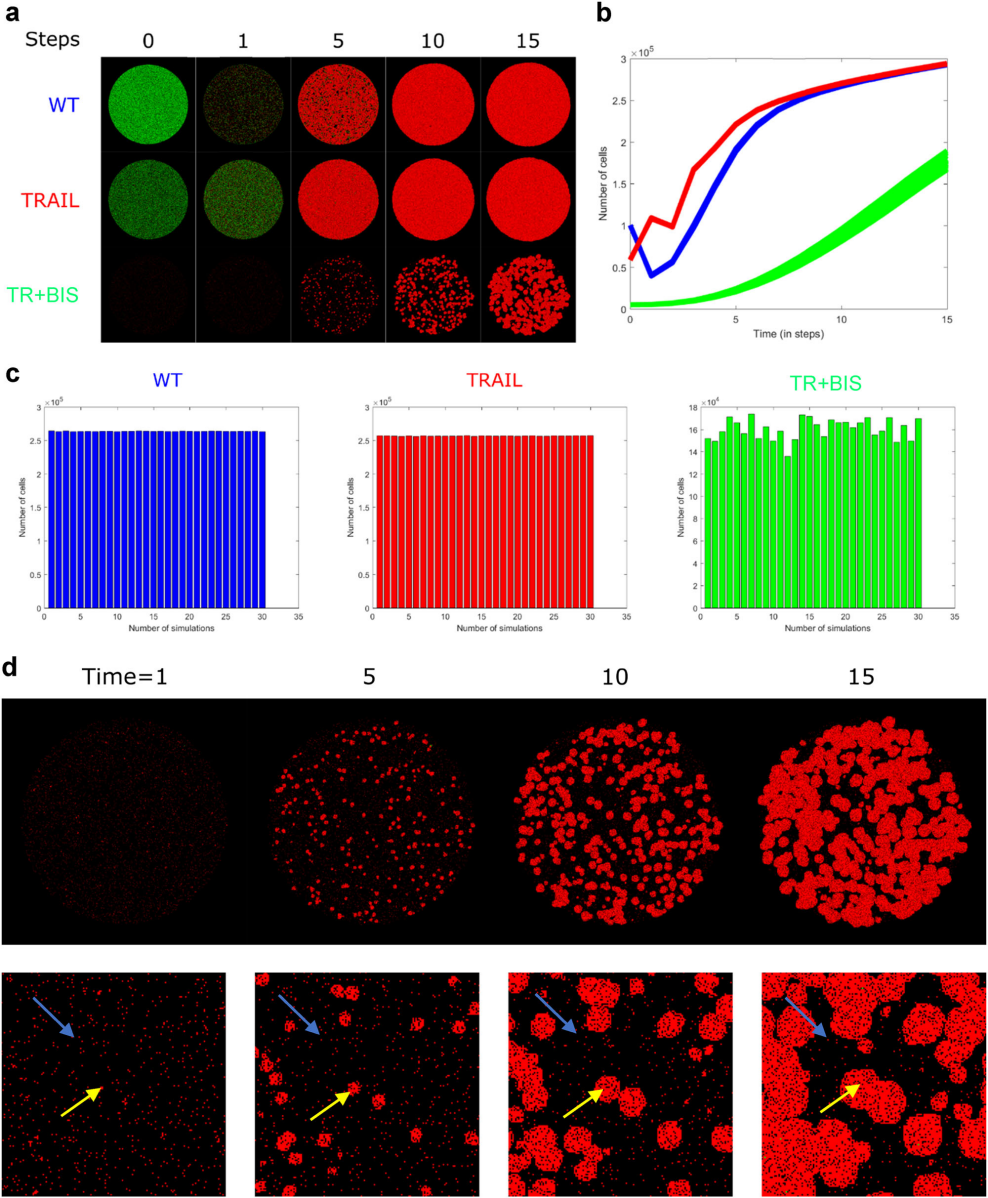
Simulations of spatiotemporal evolution of HT1080 cells using revised EMT CA model B. **a**–**c** Similar layout to Fig. 1. Note, epithelial and mesenchymal cells are represented in green and red, respectively. **d** Time evolution of isolated and clustered cells for TR + BIS Simulation. (bottom panel) Close-up views of the simulations, Blue arrow: isolated cell remains largely unaffected. Yellow arrow: cell-to-cell contact of clustered cells invokes proliferation according to CA rules

We simulated the revised CA model with actual E and M cells for the initial proportions of cells identified at time 0. For both WT and TRAIL, the resultant simulations show a general increase in the overall HT1080 population survival levels compared with the previous model without E and M cell distinction (Fig. 3a, red & blue). For TR + BIS, where each cell has a low probability of contact with another cell due to large between space, the populations remain little changed when all are E cells as observed from Fig. 1. Introducing M cells here increases the probability of contact by the moving cells. This feature allows the proliferation capacity of isolated cells through rules (ii) and (v), which this time resulted in significant cell proliferation for the TR + BIS (Fig. 3a, green).

Notably, the simulations show that at early time steps, cells that form small initial clusters through cell-cell interaction tend to grow more rapidly in time, unlike isolated cells (Fig. 3b, yellow and blue arrows). Thus, the simulations predict that even with a highly effective treatment strategy of TRAIL with BIS I for HT1080 cells, with the increased proportion of M cells relative to E cells, in the longer term, the treatment could become ineffective due to the spatial-temporal re-organization of the clustered cancers.

### Experimental investigation

**Fig. 4:**
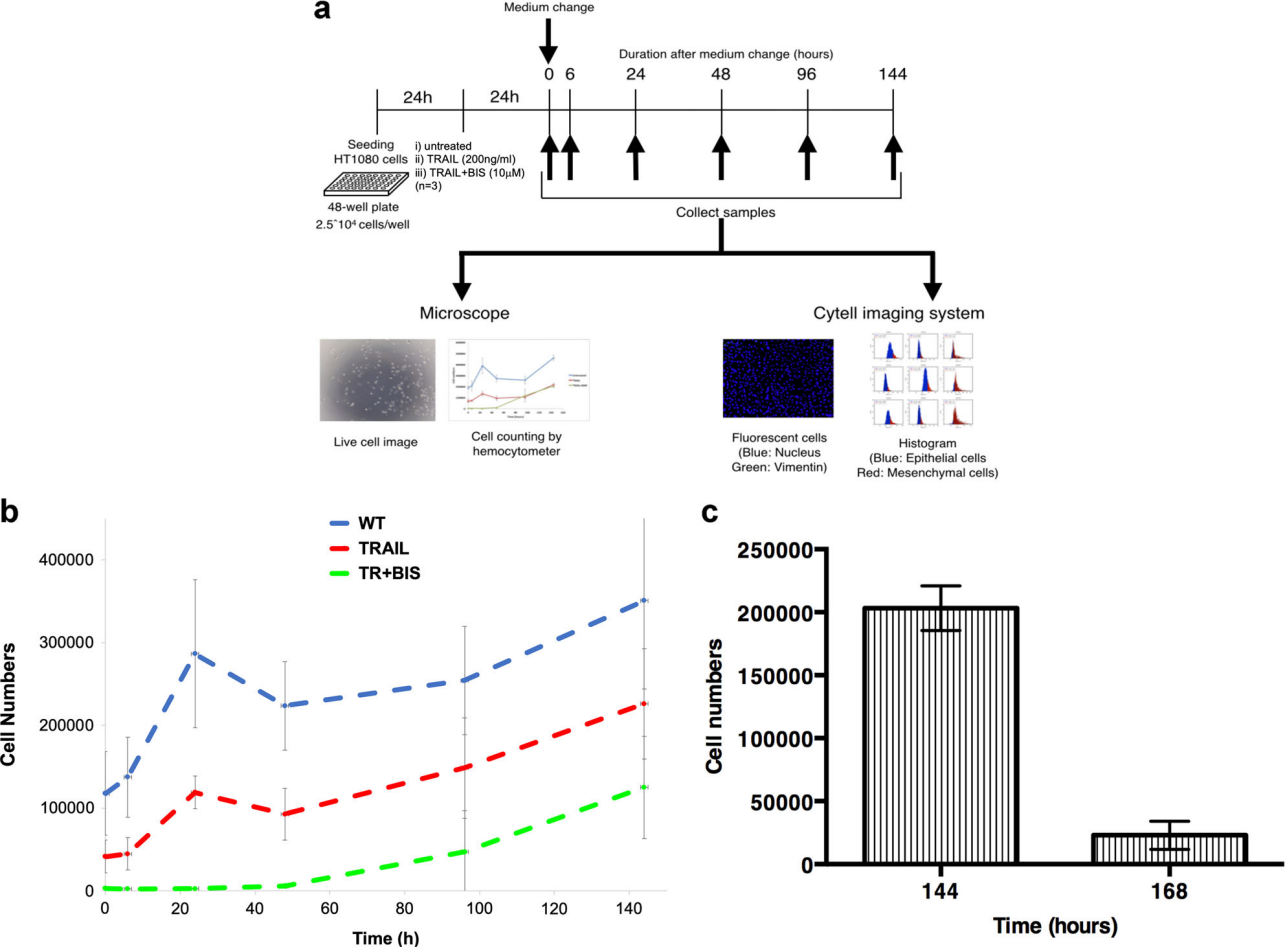
Experimental evolution of HT1080 cells. **a** Schematic experimental design. HT1080 cells were plated in 48-well plate prior to 48 h of medium change. After 24 h of plating cells, cells were treated following conditions (i) untreated (*n* = 3), (ii) TRAIL stimulated (200 ng/ml) (*n* = 3), (iii) TRAIL + BIS treated (10 μm of BIS was pre-treated 30 min prior to 200 ng/ml of TRAIL stimulation) (*n* = 3). **b** Average plot (with S.D.) of three independently repeated experiments (Fig. S3) for cell numbers against time at 0, 6, 24, 48, 96, and 144 h. Untreated in blue (WT), TRAIL-stimulated in red (TRAIL), and TRAIL + BIS in green (TR + BIS). Note that the S.D within the replicates are low (Fig. S3), but between independent runs are larger due to variable initial (*t* = 0) cancer cell numbers. **c** The effect of retreating TR + BIS for evolving HT1080 cells. HT1080 cells, treated initially with TR + BIS, were again re-treated with the same dosage of TRAIL (200 ng/ml) and BIS (10 μm) after 144 h (*n* = 3). Note that about 90% of the evolving cells died for the repeated treatment after 24 h

To scrutinize our CA predictions, we prepared time course experiments that can be directly compared with the simulation results. To monitor the proliferation of remnant HT1080 cells, we removed the existing media from the remaining cells at 24 h and replaced it with a new media without any treatment for all three conditions (Fig. 4a and Methods). The cells were monitored using live microscopy and counted at 0, 6, 24, 48, 96, and 144 h after the media were replaced.

Notably, in general accordance with our CA simulations, the HT1080 cells in (i) untreated and (ii) TRAIL-treated conditions rapidly grew and filled the entire wells within 24 h (Fig. S2–S3 and Fig. 4b). For (iii) TR + BIS treatment, the cells remained stable upto 48 h or 96 h, after which sharp proliferation occurred (Fig. S2–S3 and Fig. 4b). These data demonstrate, even after treatment that kills over 95% cells, that the remaining small number of cancer cells are able proliferate quickly after a limited lag time, and highlights the difficulty facing current therapeutics.

To check for the EMT or MET process in our cancer proliferation, we once again targeted Vimentin for fluorescence tracking in the culture with time (Methods). We noticed, for each condition, the proportion of M cells increased in time initially (indicating EMT process), but subsequently decreased when the respective cultures became overcrowded with cells for all three conditions (Fig. S2, bottom), reminiscent of MET process.^26^ Using cytell cell imaging system, this was verified by the actual counting of E and M cells’ proportions (Table 1 and Fig. S1B–F).

**Table 1:** Experimental (averaged over three replicates) proportion of epithelial (E) over mesenchymal (M) cells

Time (h)	E/M cell ratio (mean ± S.D.) %		
	WT	TR	TR + BIS
0	98.2 ± 0.4	90.6 ± 2.1	5.1 ± 1.6
6	92.8 ± 1.9	81.2 ± 1.8	4.2 ± 1.6
24	91.6 ± 0.1	91.4 ± 2.0	5.4 ± 1.2
48	81.3 ± 0.4	81.4 ± 0.3	15.2 ± 7.6
96	99.6 ± 0.1	98.5 ± 0.9	99.5 ± 0.2
144	98.5 ± 0.2	98.5 ± 0.7	98.8 ± 0.4

Finally, to test whether our TR + BIS treatment strategy will still be effective despite the self-organization of cancer cells, we specifically re-treated TR + BIS cell cultures after 144 h. Remarkably, we observed the re-treatment yielded ~ 90% cell death despite the regrowth of cells after the initial treatment (Fig. 4c). These data show that although cancer cells are able to proliferate after treatment, they do not become resistant to the second treatment within the timeframe of the experiments. Thus, we believe the cyclic treatment of TR + BIS can still be an effective long-term strategy, despite the self-organizing or proliferative ability of the remaining treated cells.

### Finalizing rules for CA Model with experimental fitting (model C)

**Fig. 5:**
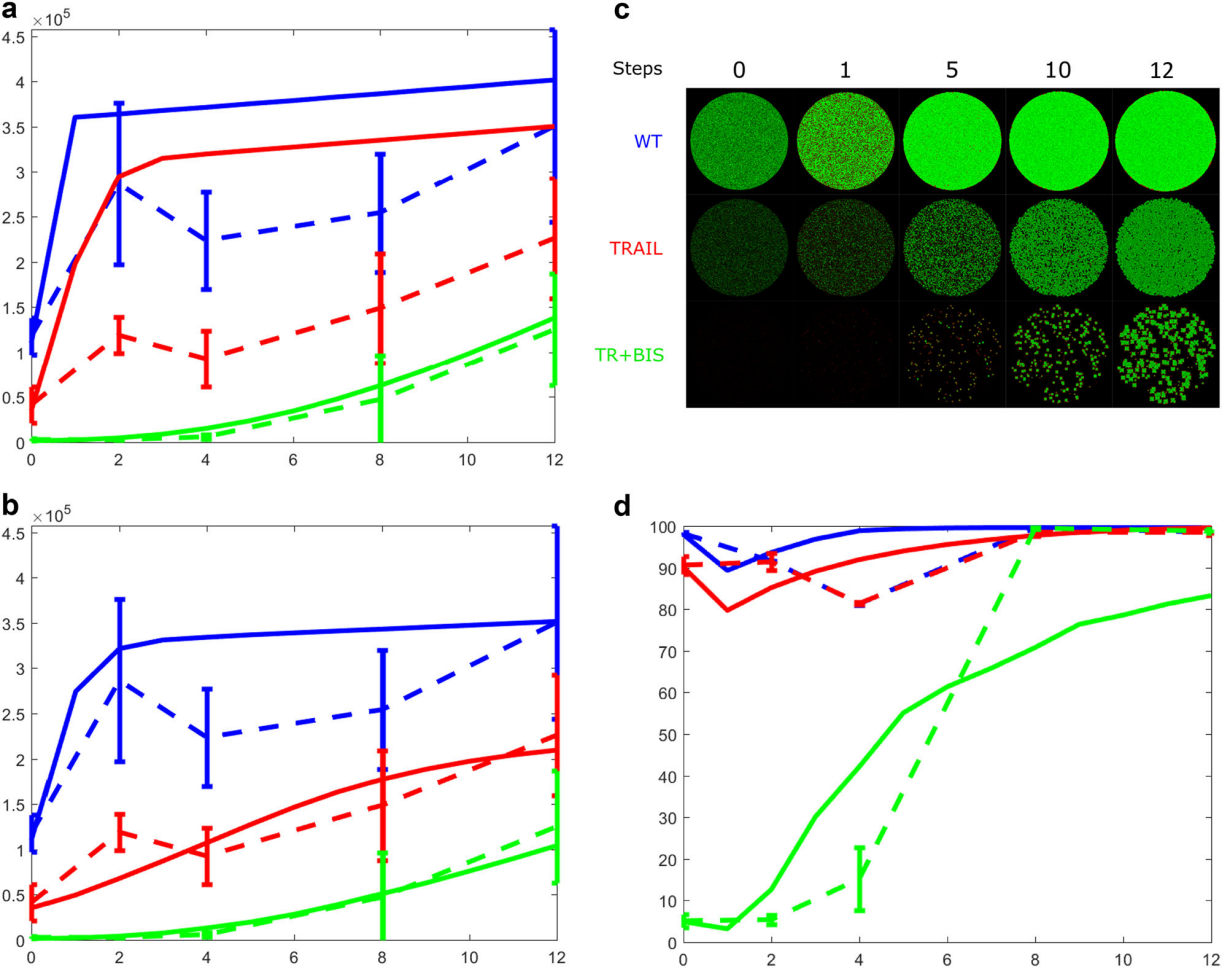
Simulations of spatiotemporal evolution of HT1080 cells using experimentally fitted model C. **a** The total numbers of cells in time (simulation in solid lines and experimental data in dashed lines with error bars). Note that this time, we kept the simulation time to 12 steps so that we cover at least two time points for every cell cycle of ~ 24 h. That is, each simulation step corresponds to 12 h or half cell cycle. **b** The total numbers of cells in time with model parameters individually fitted to each condition. The parameters for rules (i) 4 (WT), 1 (TRAIL, TR + BIS), (ii) 9 (WT), 5 (TRAIL, TR + BIS), and (iii) 5–8 (WT), 4 (TRAIL), 2–4 (TR + BIS). Simulation in solid lines and experimental data in dashed lines with error bars. **c** 2-D top view of the simulations in **b**. **d** The proportion of epithelial **e** over mesenchymal (M) cells in time. Simulation in solid lines and experimental data (Table 1) in dashed lines with error bars

Now that the experimental cancer cell proliferations have been tracked, we incorporated a genetic algorithm-based (GA) parameter fitting function into our model B and searched for a best fit using minimization of distances between experimental and simulated points (Methods). We, iteratively, modified the rules manually and allowed GA to automatically fit the cell numbers for each rule. The best result (Fig. 5a) follows:

i. Any E cell with less than four live neighbors become M cell on the next generation.
ii. Any M cell with more than eight live neighbors become E cell on the next generation.
iii. Any dead/empty cell with two to six live neighbors (E or M) becomes live cell (E or M) as by division.
iv. Any M cell is able to move randomly to an empty cell on to the next generation.
v. Any M cell that is unable to move becomes an E cell on to the next generation.

Although the simulations curves captured the general trends, WT and TRAIL cell number simulations were overpredicted for most of the time points (Fig. 5a). We conjectured that this might be owing to each condition possessing different model parameters. Thus, to better match the experimental proliferation numbers, we varied the model C parameters for rules (i) to (iii) individually to match each condition. That is, we kept the rules the same but the parameters within the rules were varied. Notably, this time, we were able to better fit the experimental data according to each condition (Fig. 5c, d).

## Discussion

In this paper, we have shown how a CA model with simple rules on cancer proliferation can be used to predict the spatial-temporal effects of cancer cells in a dish (treated or untreated). Here, it is remarkable how the complex cancer proliferations can be understood using five simple macroscopic rules. Moreover, our experiments showed that cancer cells are able to proliferate even after treatment that eradicates 95% of cells in a dish. Nevertheless, our repeated treatment still remains largely effective after cancer regrowth. In the future, it will be critical to investigate whether TRAIL + BIS treatment will successfully suppress cancer proliferations in vivo.

We also believe that the CA rules defined here may extend beyond HT1080, possibly as general principles for cancer proliferation. For example, recent experimental works on drug-resistant melanoma and patient-derived primary cells have shown the aggregation of drug resistant colonies (Fig. 1e of ref. ^30^ and Fig. 1a of ref. ^31^) similar to the clustering of cells predicted by our E & M model (Figs. 3b, 5c). Further work investigating the underlying dynamic signaling or transcriptome-wide response of such clustered resistant cells may open new doors for targeted cancer therapeutics using systems biology approaches.

## Methods

### CA model

A three-dimensional CA model (named Cancer AutoMata) was developed in Matlab code consisting of 550 × 550 × 4 cubic (1,210,000) cells, with each cell having maximum 17 neighbors for the top and bottom planes, whereas 26 neighbors for other planes. Note that the eight corners have a maximum of 7 neighbors, and 11 neighbors on the edges. However, who choose the empty initial cells large enough to avoid reaching the edges/corners within 15 time steps. We used the above initialization for lattice configuration to mimic our in vitro experiments,^11^ where cells are mainly grown in circular dishes with only a few layers of cells overlapping each other.

At time = 0 h, for each condition (Fig. 1), the cells were populated with live cells in random orientation that filled 100,000 cells for the WT, 60,000 cells for TRAIL (60% of WT) and 5000 cells for TR + BIS (5% of WT). The outcomes of different initial orientations (random distributions) of cells were tested through 30 simulations. The CA rules (see maintext) were applied from time-step 1 onwards.

The size of E and M cells were kept the same, however, M cells were able to move randomly to any available empty cell on the next generation in the three-dimensional space, using *rand* in Matlab, whereas E cells remained static throughout.

The final model C parameter values (cell numbers) were fitted using the *ga* function (https://www.mathworks.com/help/gads/ga.html). The fitness function simply minimizes the weighted distance *x* between the experimental and simulated data summed across the total simulated time. As there are five experimental time points (excluding the start point *t* = 0), *x* is summed for the five time points and minimized. The four parameters that were allowed to evolve in the model are only the cell numbers for rules 1–3 (they were allowed to vary between 0 and 26 depending on their maximum available neighbors, see above). The number of generations for fitting is automatically chosen (default is 100 generations) by the *ga* function in Matlab through the “MaxStallGenerations” in the optimization options. As this is a simple two-dimensional growth curves fitting, the total number of generations was kept with default values as the fitting convergence occurred before 100 generation.

The entire final model C, with a configuration of 550 × 550 × 2 cubic (605,000), cells is downloadable from (in matlab code with all details): https://github.com/Eclion/Cancer-AutoMata.

### Experiments

#### Reagents and cell cultures

Recombinant human TRAIL was purchased from Peprotech. BIS-I was purchased from Merck Millipore. Human fibrosarcoma cell lines (HT1080) were obtained from Japanese Collection of Research Bioresources (JCRB) cell bank. HT1080 was grown in Dulbecco's Modified Eagle's medium (DMEM, Nissui Pharmaceuticals Co., Ltd., Tokyo, Japan) supplemented with 10% fetal bovine serum, 100 U/mL penicillin, 100 mg/mL streptomycin, and 0.25 mg/mL amphotericin B at 37 °C in a humidified atmosphere with 5% CO_2_. Cells were seeded 2.5 × 10^4^ cells in each 48-well plate and incubated for 24 h.

#### Cell counting

Cells were detached with 50 µL of trypsin and suspended in DMEM, imaged and counted using microscopy and hemocytometer.

#### Imaging analysis

HT1080 were imaged using Cytell (GE Healthcare) microscope and × 10 objective lens. Cells are washed one time in phosphate-buffered saline (PBS) and fixed with 4% paraformaldehyde for 15 min. Cells were stained using mouse monoclonal anti-Vimentin antibody (Abcam) as primary antibody and diluted in PBS with Tween (PBST) at 1:500 proportion with donkey serum (1:200) for overnight at 4 ℃. Next, cells were washed in PBS three times and stained using fluorescein isothiocyanate (1:500) and donkey serum (1:200) in PBST for 2 h at room temperature. Nuclei were strained in Hoechst for 15 min at 1:250 proportions. Analysis was performed using Cytell cell imaging system software.

## Supplementary information

Suppl Figures

## Data Availability

The CA model with user instructions is found on URL: https://github.com/Eclion/Cancer-AutoMata. All raw experimental data are available from the authors.

## Supplementary information

**Supplementary Information** accompanies the paper on the *npj Systems Biology and Applications* website (10.1038/s41540-019-0084-5).
