# Supplementary material for: Defining rules for cancer cell proliferation in TRAIL stimulation
Source: NPJ Syst Biol Appl. 2019 Feb 15;5:5. doi: 10.1038/s41540-019-0084-5 (PMC6377620; doi:10.1038/s41540-019-0084-5)
Supplement: Supplementary file 1 — Suppl Figures [file 41540_2019_84_MOESM1_ESM.pdf]

Supplementary Figures

1. Histogram showing proportion of E and M cells for each condition at each time point.

(A)-(F) For each time point (0 to 144h), the numbers of E and M cells were counted for (3 independent experiments) based on the nucleus stained in blue and Vimentin stained in green. Each raw signal was detected by cytell cell imaging system. The fluorescence threshold was estimated at 0h for TRAIL+BIS treated condition (with gating parameter set to 1393), by comparing Vimentin fluorescent intensity with cell morphology.

WT WT+TR TR+BIS

(A) 0h

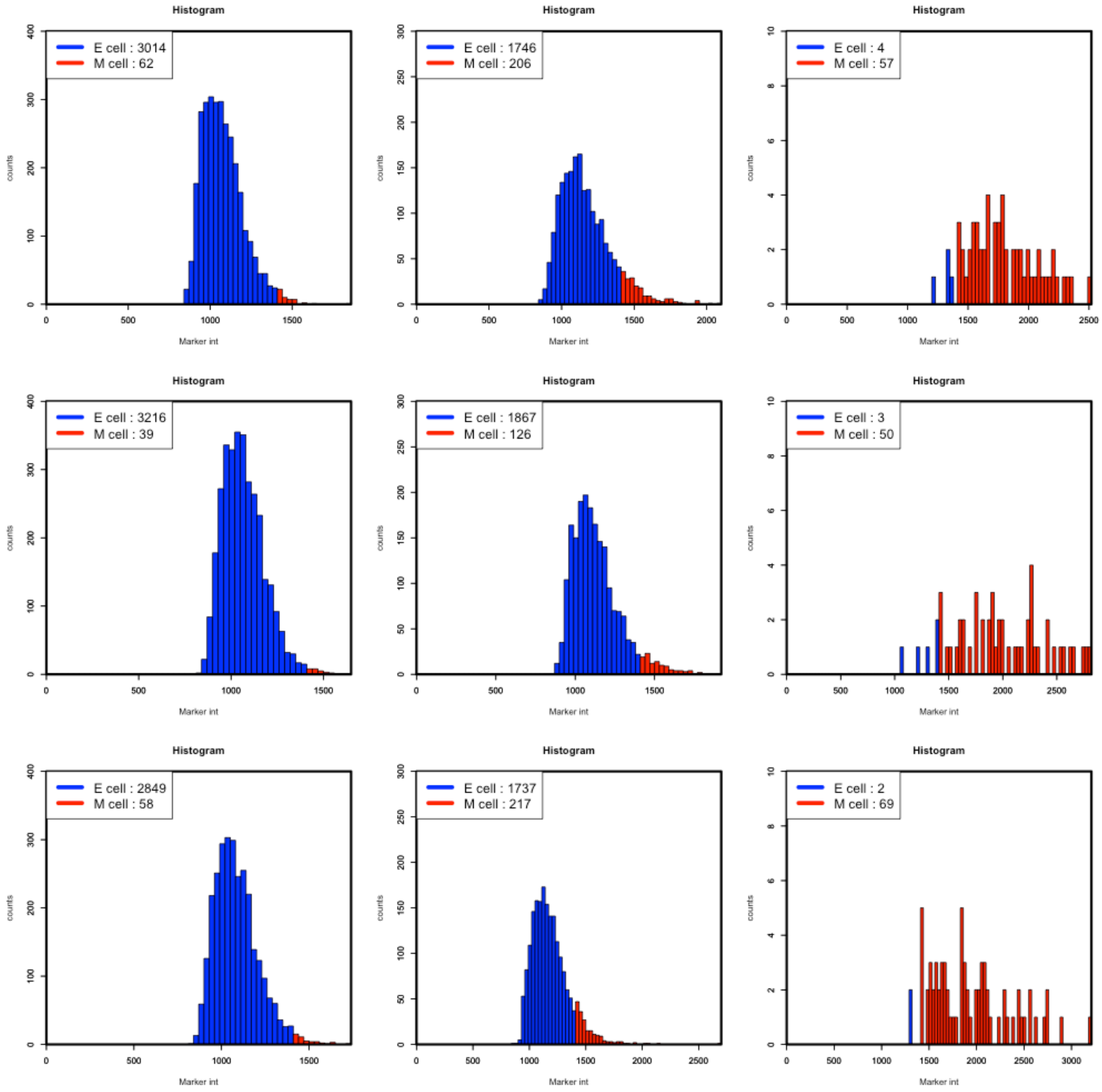

WT WT+TR TR+BIS

(B) 6h

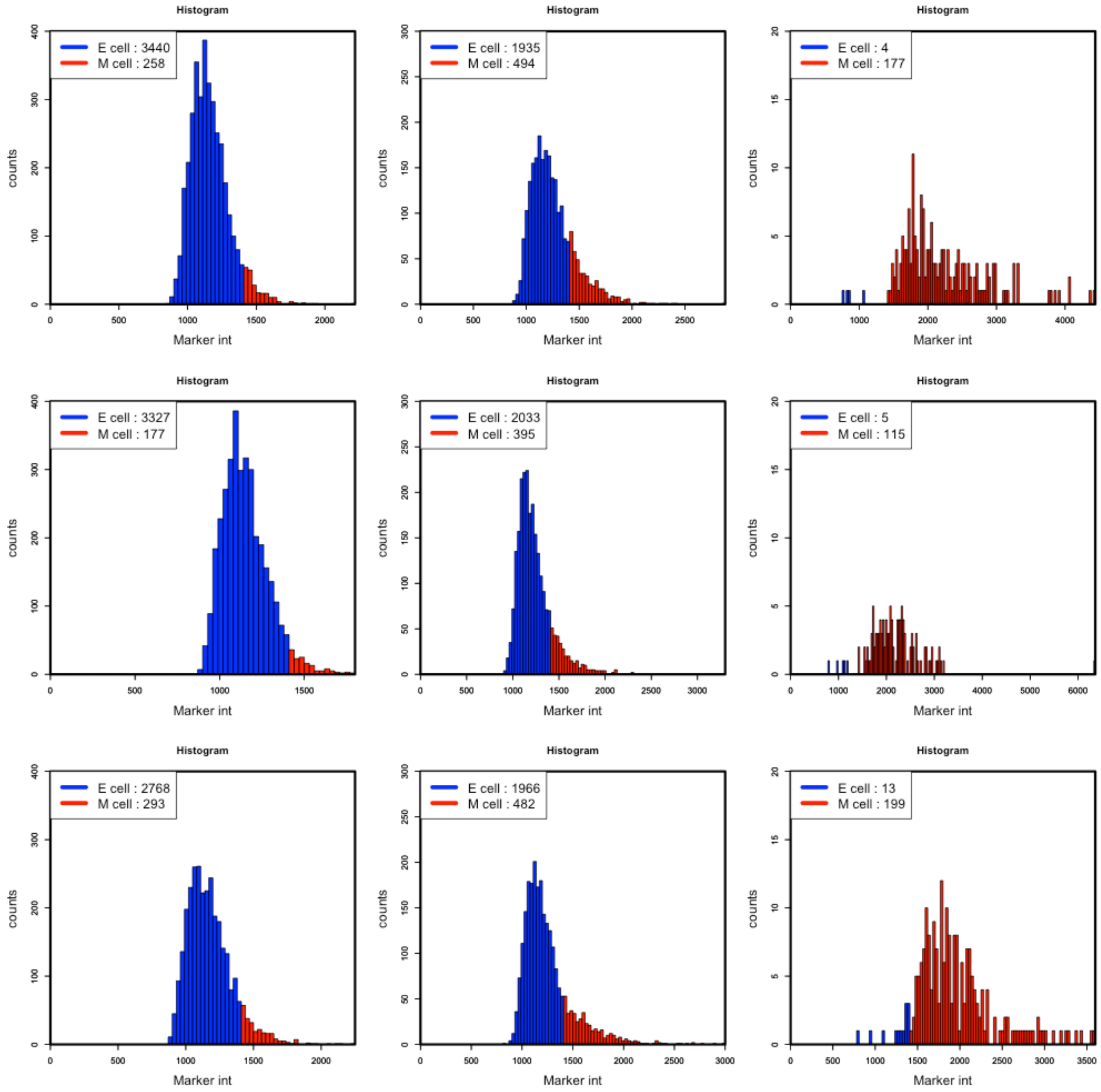

WT WT+TR TR+BIS

(C) 24h

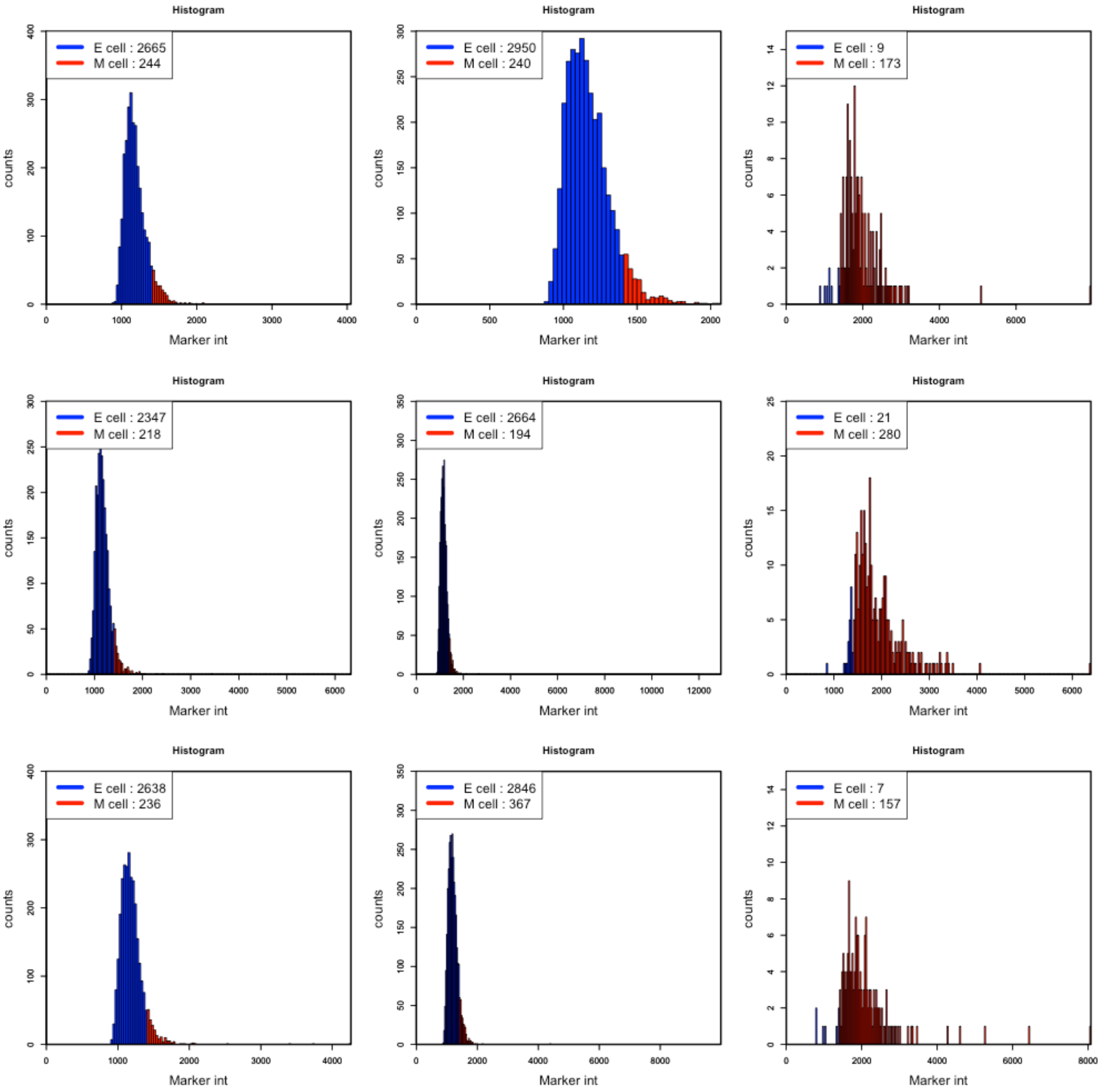

WT WT+TR TR+BIS

(D) 48h

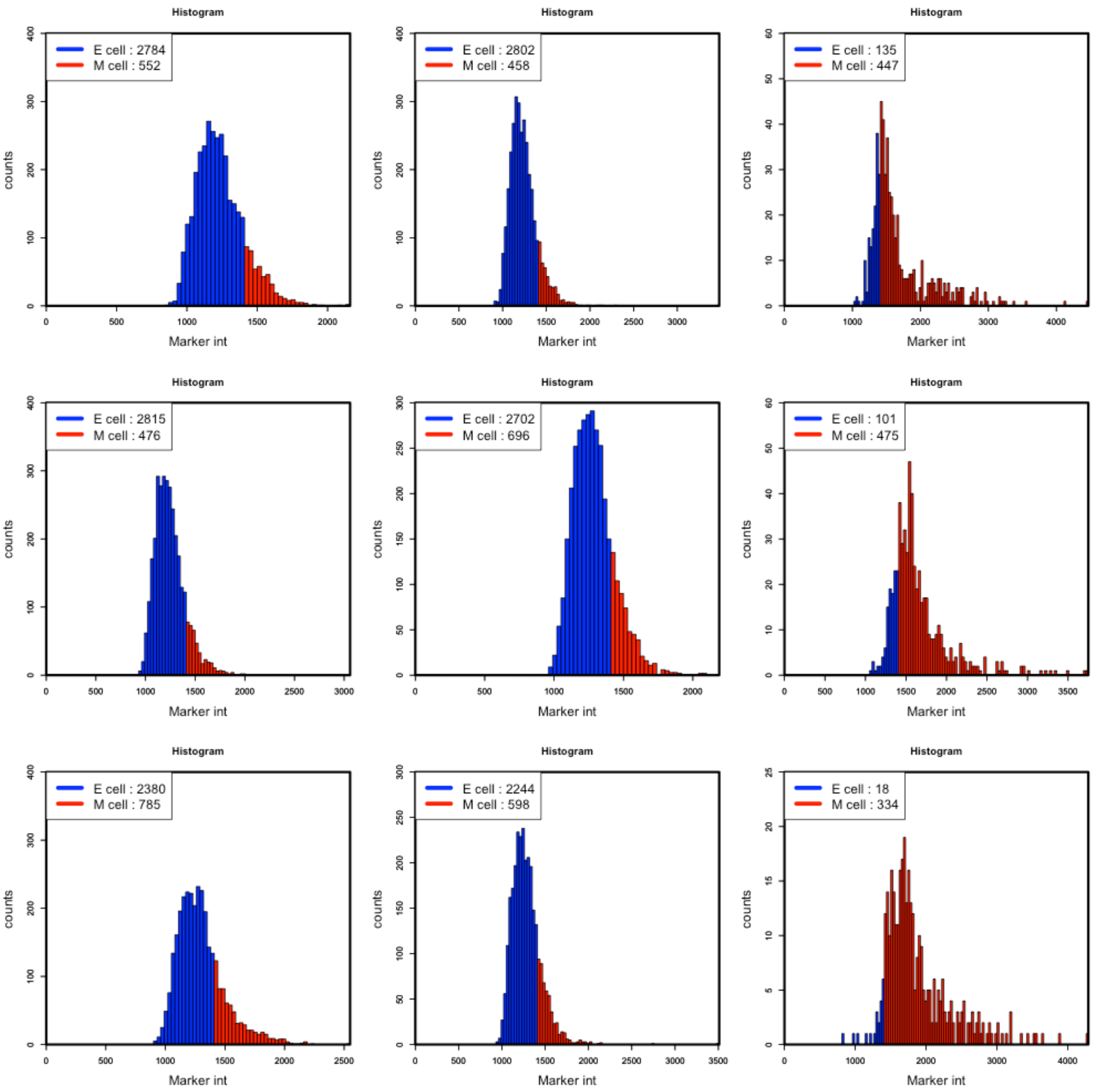

WT WT+TR TR+BIS

(A) 96h

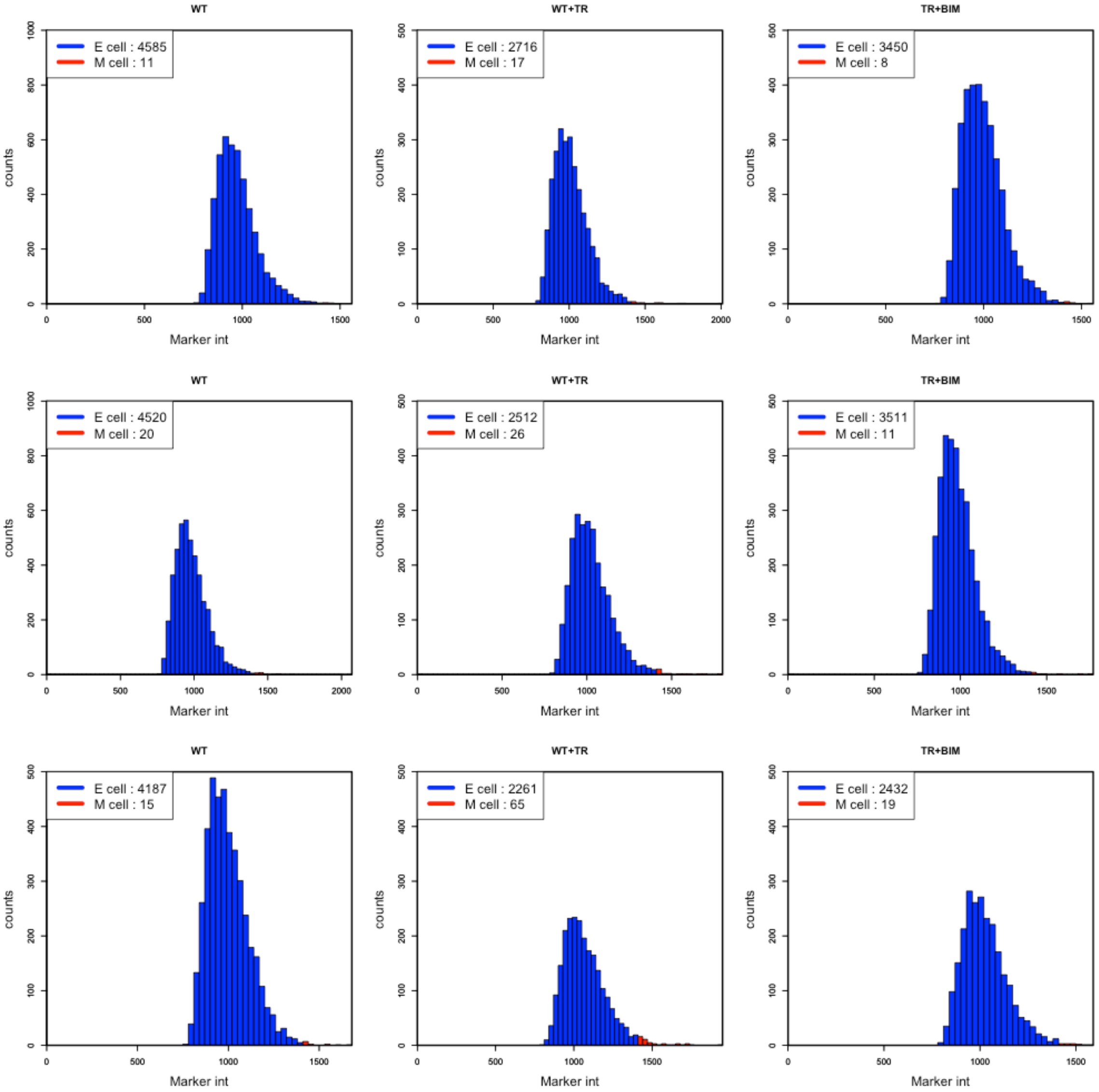

WT WT+TR TR+BIS

(A) 144h

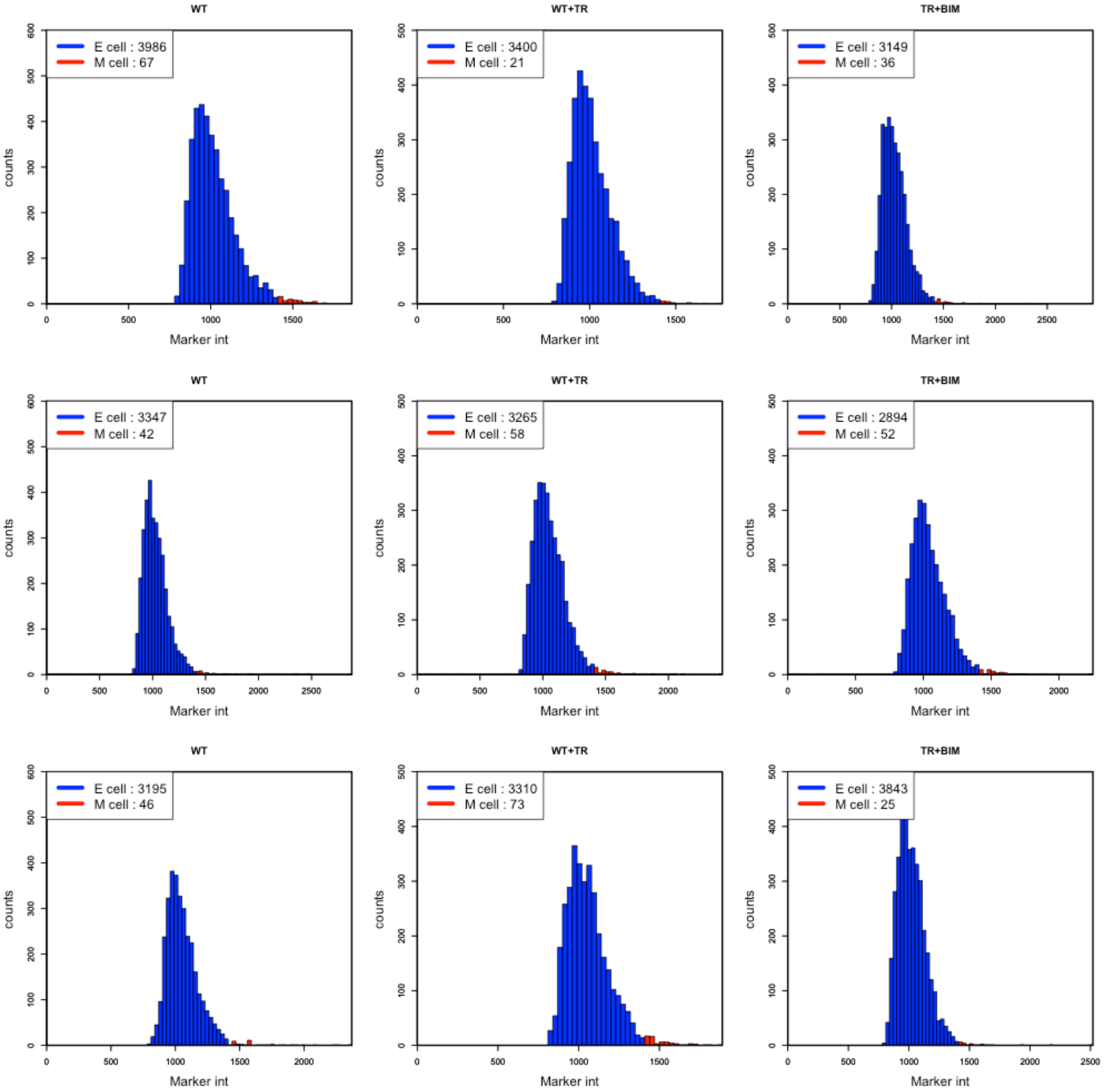

2. Fluorescence tracking of E and M cells in time.

Microscopic imaging of HT1080 cells were performed by staining cells with mouse monoclonal anti-Vimentin antibody, A) Untreated, B) TRAIL stimulated and C) TRAIL+BIS treated. Note that the regular spherical shape of E cells (stained in blue by Hoechest) is observed when cells are crowded in space for all conditions. However, for TRAIL+BIS, the cells appear thin and stretched, a hallmark of M cells, for upto 48h (marked in green by the anti-Vimentin antibody). Dark images, especially in 96 and 144h in (A), (B) and (C), are a result of the cell population crowding.

A) Untreated

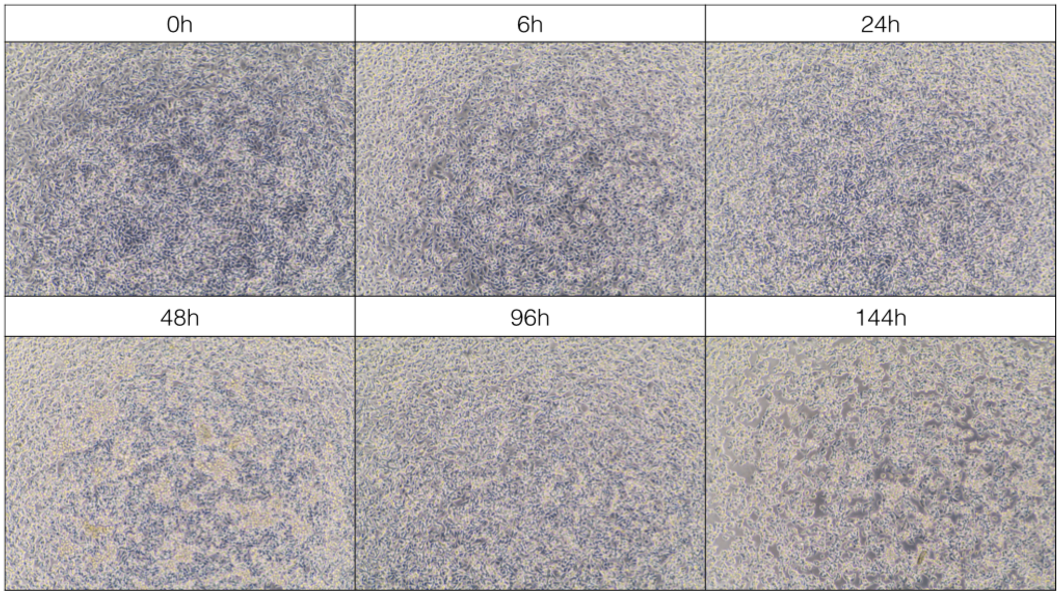

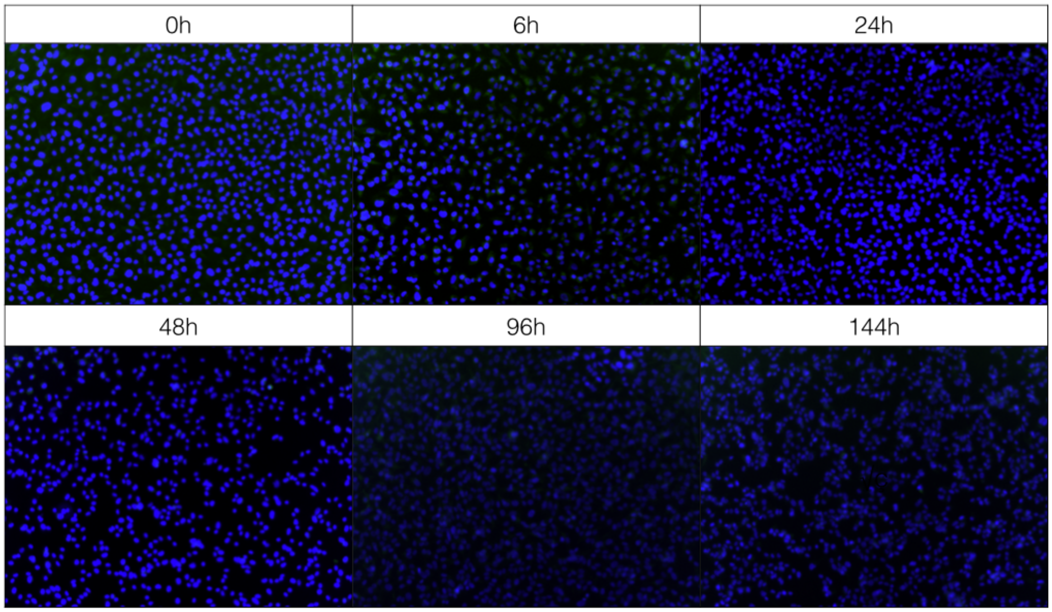

B) TRAIL

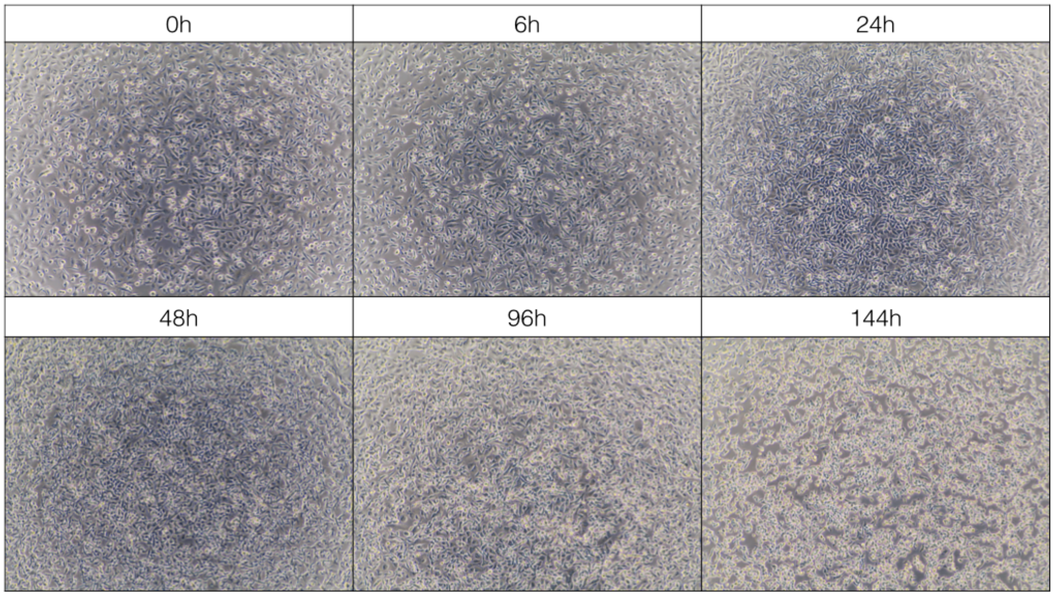

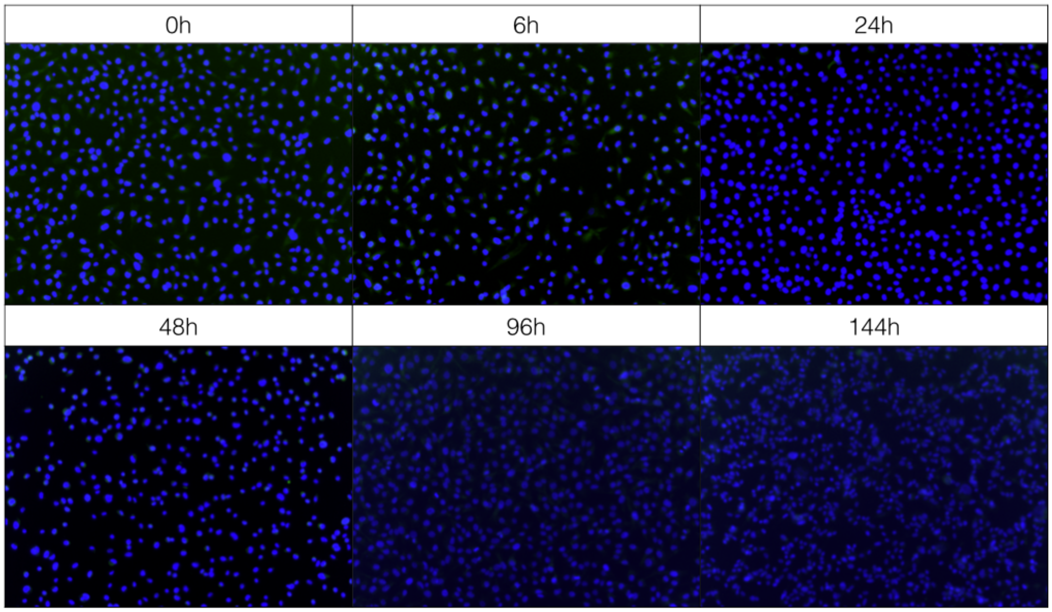

C) TRAIL+BIS

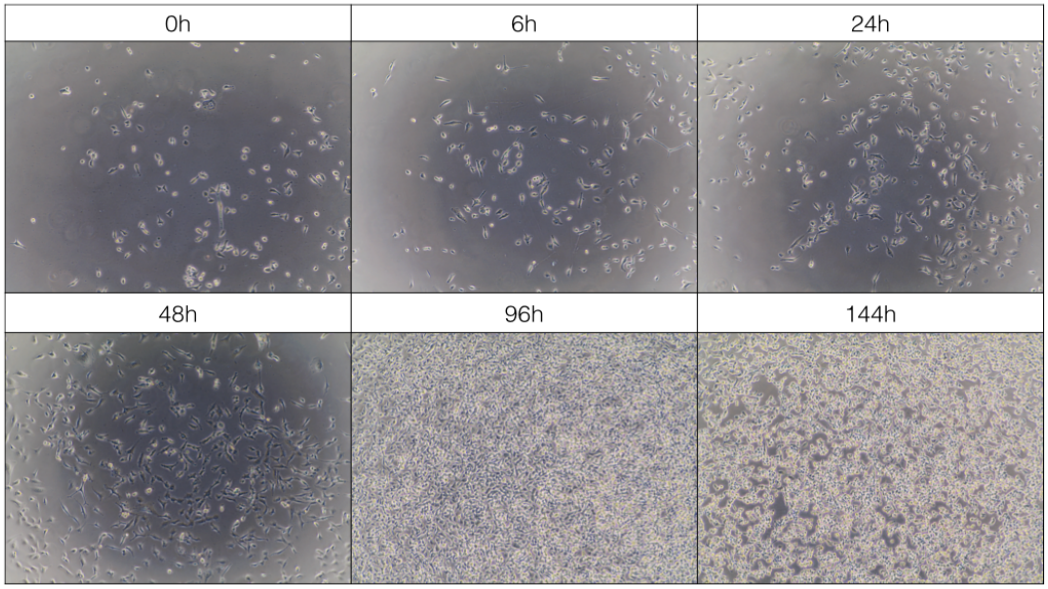

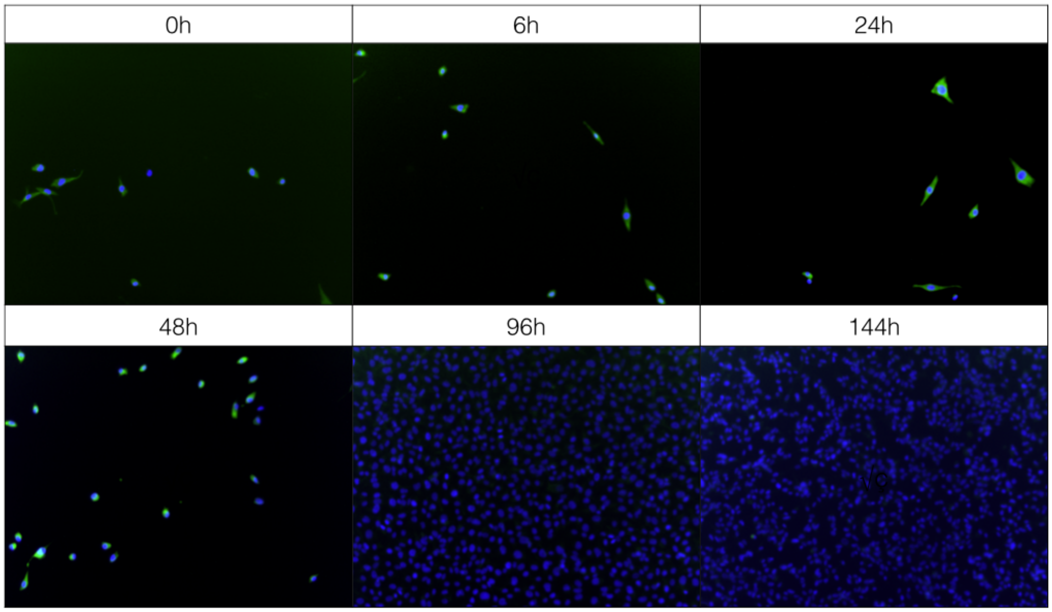

3. Experimental evolution of HT1080 cells.

Plots of 3 independent repeat experiments for cell numbers (n = 3, replicates for each experiment) against time at 0, 6, 24, 48, 96 and 144h. Untreated in blue, TRAIL stimulated in red, and TRAIL+BIS in green.

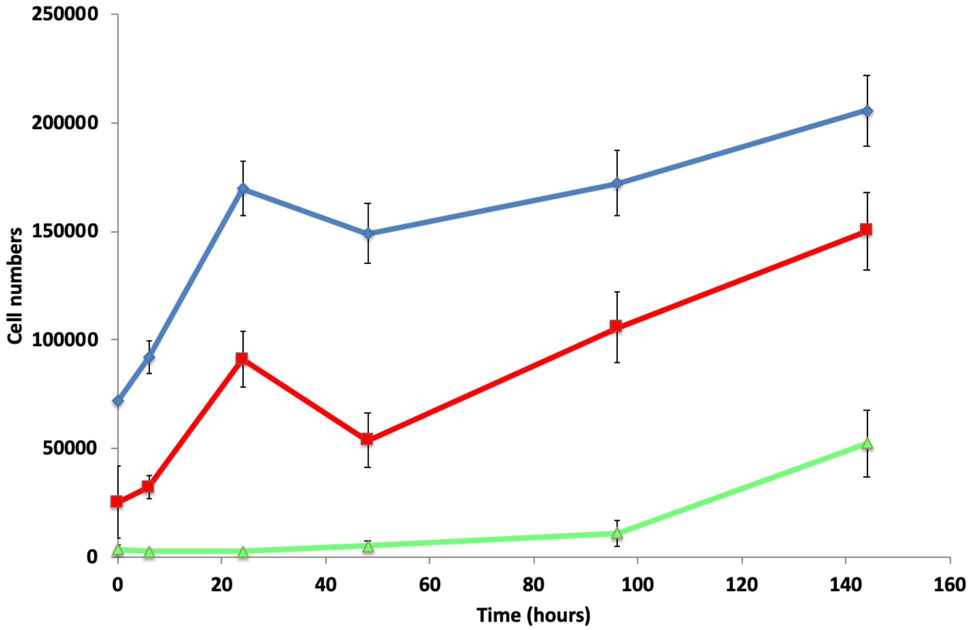

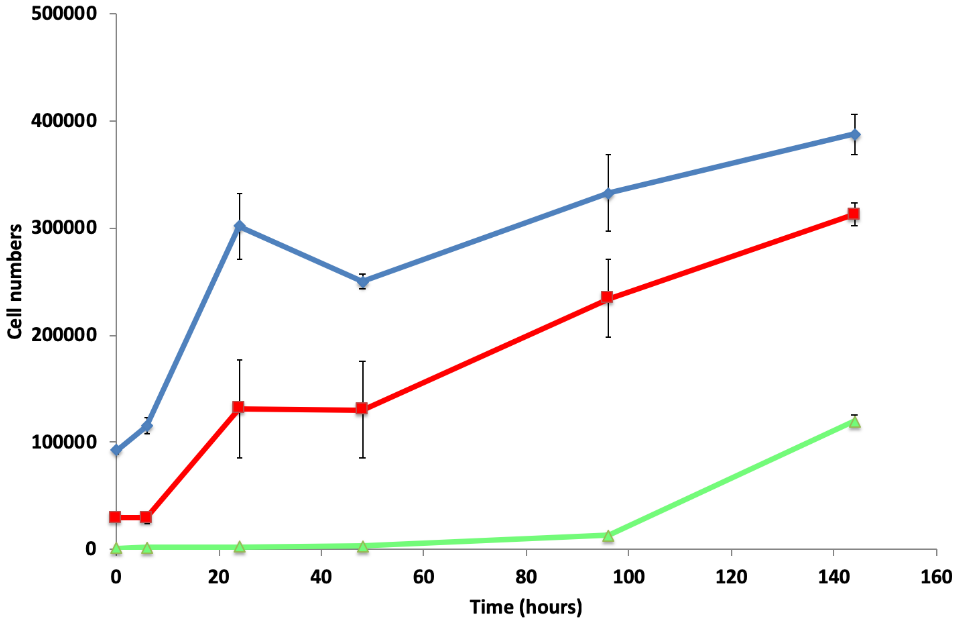

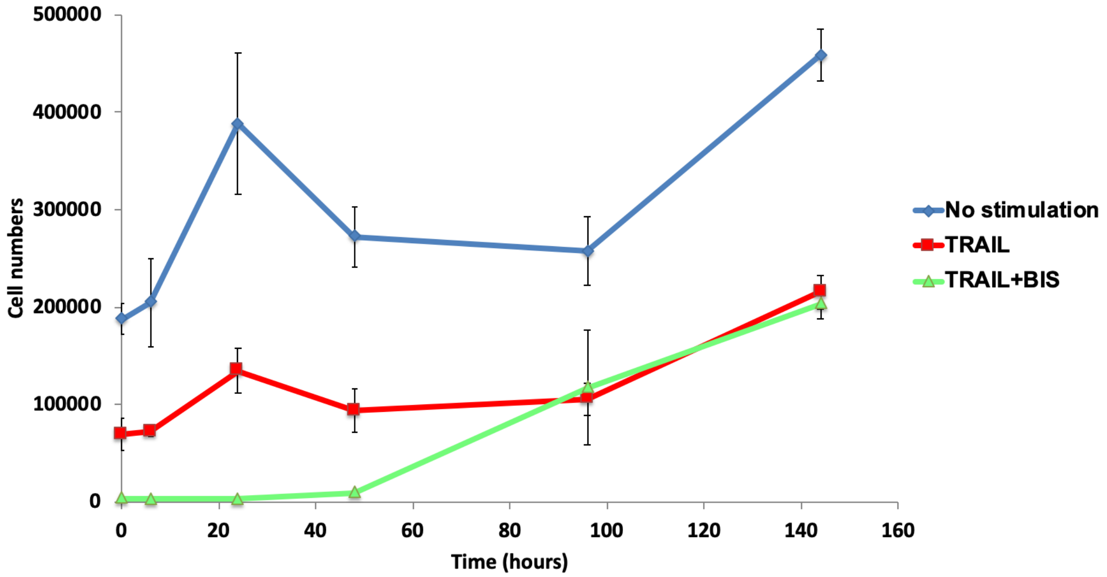
500000
400000
300000
200000
100000
0
Cell numbers
0
20
40
60
80
100
120
140
160
Time (hours)
No stimulation
TRAIL
TRAIL+BIS
